# Hybrid High-Power AlGaN/CdZnO/GaN/AlGaN HEMT with High Breakdown Voltage

**DOI:** 10.3390/ma17225560

**Published:** 2024-11-14

**Authors:** Bonghwan Kim, Seung-Hwan Park

**Affiliations:** Department of Semiconductor Electronic Engineering, Daegu Catholic University, Gyeongsan 38430, Gyeongbuk, Republic of Korea; shpark@cu.ac.kr

**Keywords:** HEMT, TCAD, GaN, CdZnO, breakdown voltage, transconductance, high-power electronics

## Abstract

This study investigates the effects of incorporating a CdZnO layer in place of the conventional InGaN layer in an AlGaN/InGaN/GaN/AlGaN/SiC high-electron mobility transistor (HEMT) structure. We examine the resulting characteristics and assess the potential of high-power HEMT applications, including high-power switching converters, through simulation analysis. Both structures demonstrate increased drain current and transconductance with increasing Al content in the barrier layer. However, HEMTs with a CdZnO layer exhibit higher drain current compared to those with an InGaN layer at the same Al content. The breakdown voltage decreases rapidly with increasing Al content, attributed to changes in electric field distribution. HEMTs with a CdZnO/GaN channel exhibit a slightly higher breakdown voltage (~795 V) compared to those with an InGaN/GaN channel (~768 V) at a lower Al content of x = 0.10. These results suggest that CdZnO-based HEMTs have significant potential for high-power, high-frequency applications.

## 1. Introduction

High-electron mobility transistors (HEMTs) based on gallium nitride (GaN) have emerged as promising candidates for high-power and high-frequency applications in recent years. These devices exhibit superior characteristics such as high breakdown voltage, high electron saturation velocity, and excellent thermal conductivity, making them particularly suitable for power electronics, wireless communication systems, and radar applications [1,2,3,4,5]. The continuous demand for improved performance in these fields has driven researchers to explore novel HEMT structures and materials [1,2,3,4,5,6,7,8,9,10,11].

One of the critical parameters in HEMT design is the breakdown voltage, which directly impacts the device’s power handling capability and reliability [12,13,14,15,16]. Various strategies have been employed to enhance the breakdown voltage, including the incorporation of field plates, optimization of buffer layers, and the use of novel material combinations. Among these approaches, the insertion of back-barrier layers has shown promising results in improving the confinement of the two-dimensional electron gas (2DEG) and, consequently, the overall device performance.

Recent advancements in material science have led to the exploration of hybrid HEMT structures incorporating zinc oxide (ZnO)-related compounds. These materials offer unique properties that could potentially complement or enhance the characteristics of traditional GaN-based HEMTs. However, research in this area remains in its early stages, and many fundamental aspects of these hybrid structures have yet to be fully understood [17,18,19,20,21].

In this study, we investigate the effects of incorporating a cadmium zinc oxide (CdZnO) layer in place of the conventional indium gallium nitride (InGaN) layer within an AlGaN/InGaN/GaN/AlGaN/SiC HEMT structure. Through comprehensive simulations, we analyze the impact of this structural modification on key device parameters, with a particular focus on its potential for high-power applications. Our work aims to contribute to the growing body of knowledge on hybrid HEMT structures and explore new avenues for performance enhancement in GaN–based power devices.

The remainder of this paper is organized as follows: Section 2 describes the simulation methodology and device structures under investigation. Section 3 present the results of our simulations and discuss the observed effects of the CdZnO layer on device characteristics. Finally, Section 4 concludes the paper with a summary of our findings and suggestions for future research directions. The characteristics of the device were simulated using the Silvaco technology computer-aided design (TCAD, Silvaco 5.2.23.R) simulation tool [22].

## 2. Methods

The device characteristics were calculated using the Silvaco TCAD simulation tool. Two HEMT structures were simulated: (1) a conventional AlGaN/InGaN/GaN/AlGaN/SiC HEMT with an InGaN/GaN coupling channel, and (2) a hybrid AlGaN/CdZnO/GaN/AlGaN/SiC HEMT with a CdZnO/GaN coupling channel.

The devices were built on a silicon carbide (4H–SiC) substrate with the following layer structure:

- 1 μm thick Al_0.05_Ga_0.95_N back–barrier layer
- 10 nm thick GaN layer
- 10 nm thick In0.1Ga0.9N layer (or Cd_0.1_Zn_0.9_O layer for the hybrid structure)
- 10 nm thick Al_x_Ga_1−x_N barrier layer (x varied from 0.1 to 0.3)
- 500 nm Silicon nitride (SiN) passivation layer

Gaussian-distributed donor doping of 1 × 10^18^ cm^−3^ was applied near both electrodes of the coupling channel. The gate length was set to 2 μm. The Al content in the barrier AlGaN layer was varied from 0.1 to 0.3 to investigate its effect on device performance. 

**Figure 1:**
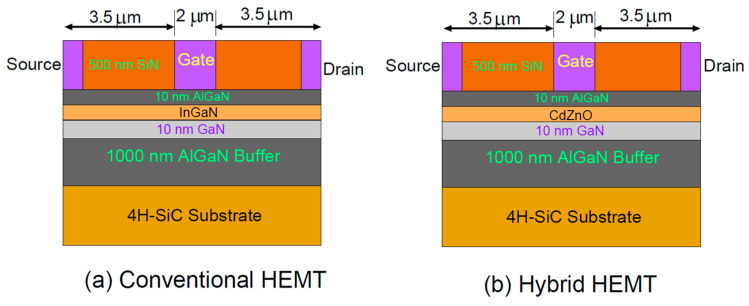
(**a**) The conventional AlGaN/InGaN/GaN/AlGaN/SiC HEMT structure with an InGaN/GaN coupling channel, and (**b**) the hybrid AlGaN/CdZnO/GaN/AlGaN/SiC HEMT structure with a CdZnO/GaN coupling channel.

Figure 1 shows (a) the conventional AlGaN/InGaN/GaN/AlGaN/SiC HEMT structure with an InGaN/GaN coupling channel, and (b) the hybrid AlGaN/CdZnO/GaN/AlGaN/SiC HEMT structure with a CdZnO/GaN coupling channel.

## 3. Results and Discussion

**Figure 2:**
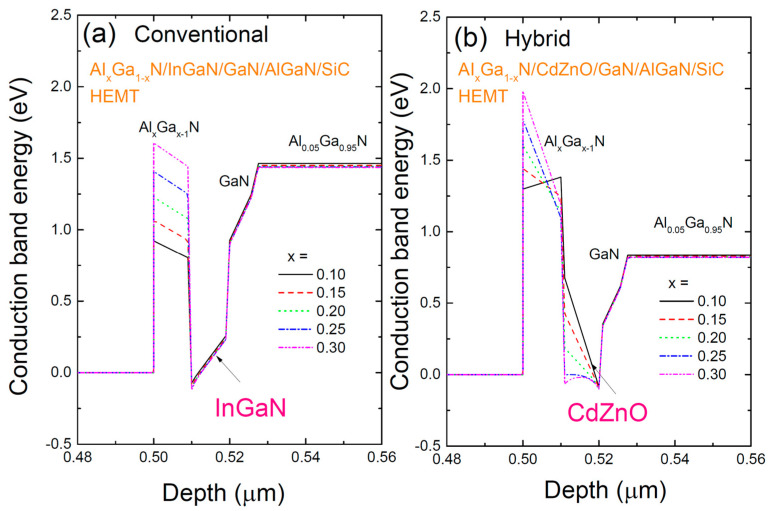
The conduction band profiles of two structures: (**a**) the conventional AlGaN/InGaN/GaN/AlGaN/SiC HEMT with an InGaN/GaN coupling channel, and (**b**) a hybrid AlGaN/CdZnO/GaN/AlGaN/SiC HEMT with a CdZnO/GaN coupling channel, with varying Al content in the AlGaN barrier.

Figure 2 shows the conduction band profiles of two structures: (a) the conventional AlGaN/InGaN/GaN/AlGaN/SiC HEMT with an InGaN/GaN coupling channel, and (b) a hybrid AlGaN/CdZnO/GaN/AlGaN/SiC HEMT with a CdZnO/GaN coupling channel, with varying Al content in the AlGaN barrier. The addition of materials with smaller bandgaps, such as InGaN and CdZnO, deepens the potential well, creating a triangular shape. The HEMT with an InGaN layer shows that the well shape is almost unaffected by the Al content in the barrier, except that increasing the Al content increases the barrier height due to the larger bandgap. On the other hand, in the HEMT with a CdZnO layer, as the Al content increases, the well shape transitions from triangular to U-shaped, increasing the spatial confinement of the electron wave function and thus enhancing the electron concentration within the well. This change can be attributed to the larger spontaneous polarization of CdZnO (−0.099 C/m²) compared to InGaN (−0.032 C/m²), causing a shift in the potential due to the polarization field. In the case of the HEMT with the InGaN layer, there is little change in the internal field in the barrier. However, in the HEMT with the CdZnO layer, as the Al content increases, the polarization difference between the well and the barrier decreases rapidly, flattening the well shape.

**Figure 3:**
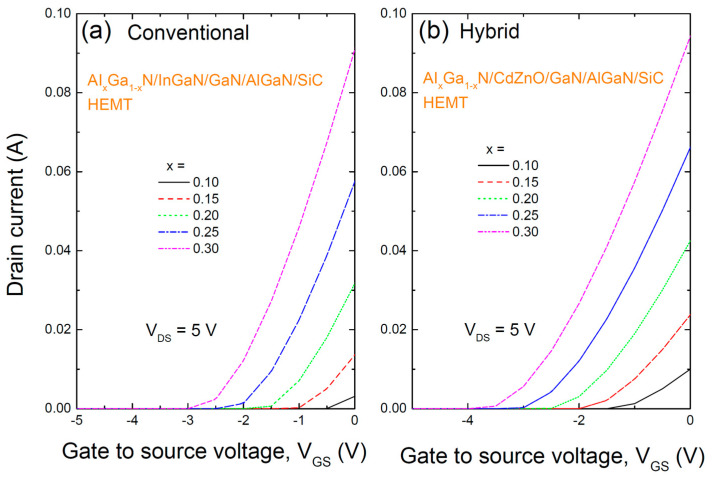
The transfer characteristics of two HEMT structures: (**a**) the conventional AlGaN/InGaN/GaN/AlGaN/SiC HEMT with an InGaN/GaN coupling channel, and (**b**) a hybrid AlGaN/CdZnO/GaN/AlGaN/SiC HEMT with a CdZnO/GaN coupling channel, with varying Al content in the AlGaN barrier.

Figure 3 shows the transfer characteristics of two HEMT structures: (a) the conventional AlGaN/InGaN/GaN/AlGaN/SiC HEMT with an InGaN/GaN coupling channel, and (b) a hybrid AlGaN/CdZnO/GaN/AlGaN/SiC HEMT with a CdZnO/GaN coupling channel, with varying Al content in the AlGaN barrier. The gate voltage V_GS_ was varied from −5 to 0 V, and the drain voltage V_DS_ was fixed at 5 V. As the Al content increases, both structures exhibit an increase in the drain current at a given gate voltage, as well as a rise in the threshold voltage. The HEMT with the CdZnO layer demonstrates a higher threshold voltage. Although not explicitly shown, the subthreshold swing (SS) for the CdZnO-based HEMT is lower compared to the InGaN-based HEMT, as indicated by the calculated values. For example, at x = 0.25, the SS for the CdZnO-based HEMT is approximately 290 mV/dec, compared to about 500 mV/dec for the InGaN-based HEMT. A lower SS is preferable for HEMTs.

**Figure 4:**
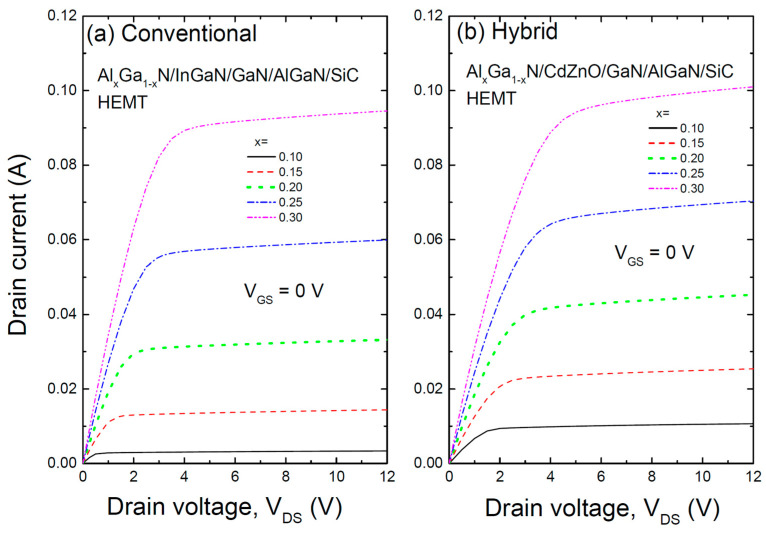
The I–V characteristics of two HEMT structures: (**a**) the conventional AlGaN/InGaN/GaN/AlGaN/SiC HEMT with an InGaN/GaN coupling channel, and (**b**) a hybrid AlGaN/CdZnO/GaN/AlGaN/SiC HEMT with a CdZnO/GaN coupling channel, calculated at zero gate voltage.

Figure 4 shows the I–V characteristics of two HEMT structures: (a) the conventional AlGaN/InGaN/GaN/AlGaN/SiC HEMT with an InGaN/GaN coupling channel, and (b) a hybrid AlGaN/CdZnO/GaN/AlGaN/SiC HEMT with a CdZnO/GaN coupling channel, calculated at zero gate voltage. High output current density I_DS,max_ and high breakdown voltage VBR are essential for achieving high output power density in RF power amplifiers. In both cases, as the Al content increases, the drain current increases. For the HEMT with the InGaN layer, this is explained by the deeper potential well due to increased Al content, which enhances electron confinement. In the HEMT with the CdZnO layer, the potential well transitions from triangular to U-shaped as the Al content increases, resulting in even greater current than in the InGaN-based HEMT at the same Al content due to the increased electron confinement in the U-shaped well.

**Figure 5:**
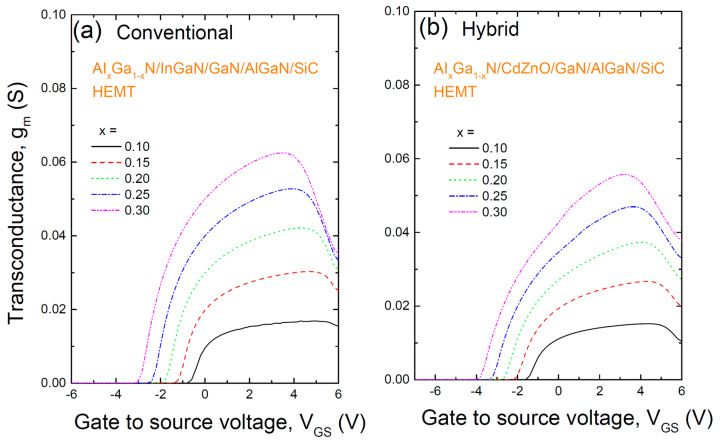
The transconductance (gm) of two HEMT structures: (a) the conventional AlGaN/InGaN/GaN/AlGaN/SiC HEMT with an InGaN/GaN coupling channel, and (b) a hybrid AlGaN/CdZnO/GaN/AlGaN/SiC HEMT with a CdZnO/GaN coupling channel, with varying Al content in the AlGaN barrier.

Figure 5 shows the transconductance (g_m_) of two HEMT structures: (a) the conventional AlGaN/InGaN/GaN/AlGaN/SiC HEMT with an InGaN/GaN coupling channel, and (b) a hybrid AlGaN/CdZnO/GaN/AlGaN/SiC HEMT with a CdZnO/GaN coupling channel, with varying Al content in the AlGaN barrier. Transconductance measures the change in current relative to the change in gate voltage and is defined as g_m_ = ∂I_DS_/∂V_GS_. A high transconductance is essential for high-speed HEMT operation and enhances gate transfer efficiency. Transconductance increases with increasing gate voltage until it reaches a peak, after which it drops sharply—a phenomenon that is a major issue in nanoscale HEMTs. The gate voltage swing (GVS) is defined as the gate bias range within 80% of the peak gm. The calculated GVS is slightly smaller for the CdZnO-based HEMT but still comparable to the InGaN-based HEMT, with values of approximately 4.8 V and 5.0 V, respectively, at x = 0.25. A high GVS is essential for high linearity radio frequency (RF) applications. Both HEMT structures show an increase in transconductance with increasing Al content. While the transconductance values are similar at lower Al content, the InGaN-based HEMT shows a slightly higher transconductance at higher Al content. For example, at x = 0.25, the CdZnO-based HEMT has a gm of about 0.047 S (for a gate length of 2 µm), compared to about 0.053 S for the InGaN-based HEMT.

**Figure 6:**
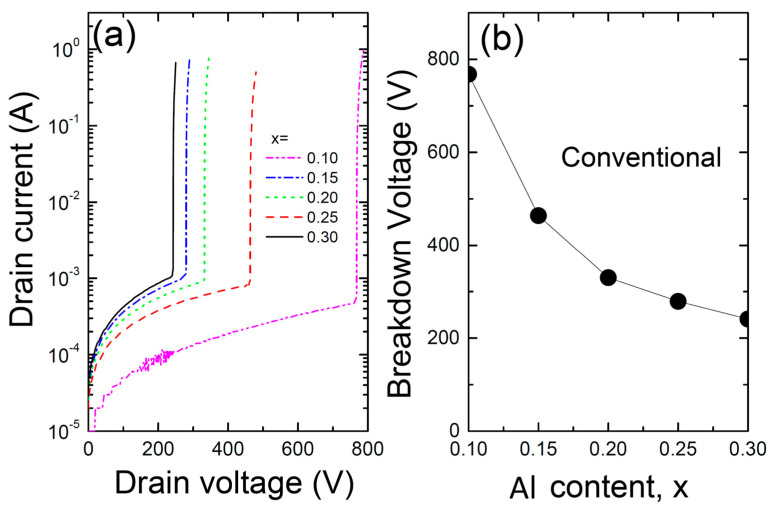
(**a**) The relationship between drain voltage and current, and (**b**) the breakdown voltage V_BR_ as a function of Al content for the conventional AlGaN/InGaN/GaN/AlGaN/SiC HEMT with an InGaN/GaN coupling channel.

Figure 6 shows (a) the relationship between drain voltage and current and (b) the breakdown voltage V_BR_ as a function of Al content for the conventional AlGaN/InGaN/GaN/AlGaN/SiC HEMT with an InGaN/GaN coupling channel. In the HEMT simulations, Selberherr’s impact ionization model was used [23,24,25,26,27]. The breakdown voltage is highly dependent on the Al content in the barrier, showing a rapid decrease followed by saturation as the Al content increases. While the exact reason for this behavior remains unclear, it is speculated that lower Al content results in a more uniform electric field distribution, thus increasing the breakdown voltage. However, further investigation is needed. To increase the breakdown voltage, a barrier with low Al content is recommended, although this could lead to electron confinement issues, reducing drain current as shown in Figure 3. Therefore, optimization is necessary depending on the device’s purpose. As the Al content increases, the electron gas density at the AlGaN/InGaN interface also rises. Consequently, this increase in electron density leads to a stronger electric field concentration at high voltages, resulting in a lower breakdown voltage for devices with higher Al content.

**Figure 7:**
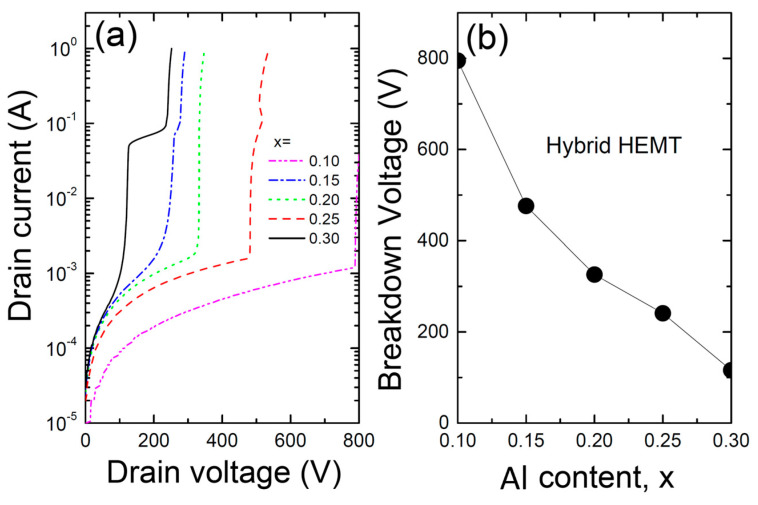
(**a**) The relationship between drain voltage and current, and (**b**) the breakdown voltage V_BR_ as a function of Al content for the hybrid AlGaN/CdZnO/GaN/AlGaN/SiC HEMT with a CdZnO/GaN coupling channel.

Figure 7 shows (a) the relationship between drain voltage and current and (b) the breakdown voltage V_BR_ as a function of Al content for the hybrid AlGaN/CdZnO/GaN/AlGaN/SiC HEMT with a CdZnO/GaN coupling channel. The breakdown voltage for the CdZnO-based HEMT also depends heavily on the Al content, decreasing rapidly with increasing Al content. Similarly to the InGaN/GaN channel, this is likely due to the uniformity of the electric field distribution at lower Al content, though more research is needed to confirm this. As in the previous case, lower Al content increases the breakdown voltage but can reduce electron confinement, leading to a decrease in drain current. However, the CdZnO-based HEMT shows a slightly higher breakdown voltage than the InGaN-based HEMT at the lower Al content (x = 0.10).

**Figure 8:**
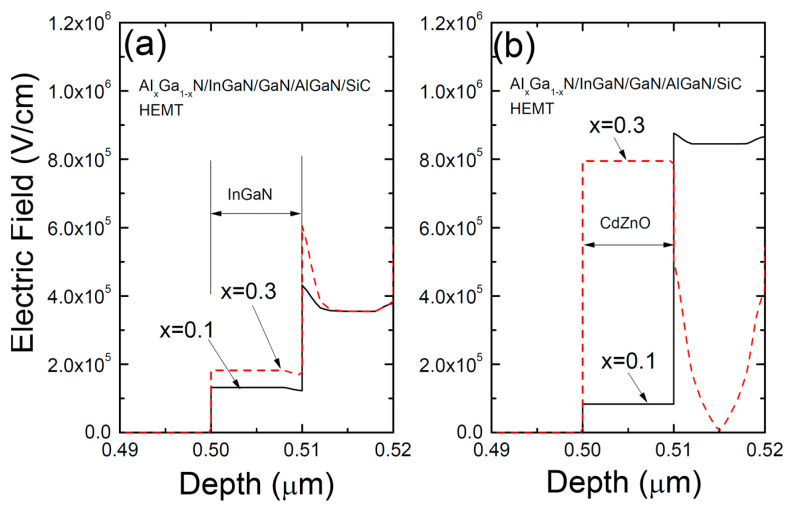
The electric field (V/cm) along a cutline through the center of the gate region for two structures: (**a**) a conventional AlGaN/InGaN/GaN/AlGaN/SiC HEMT with an InGaN/GaN coupling channel, and (**b**) a hybrid AlGaN/CdZnO/GaN/AlGaN/SiC HEMT with a CdZnO/GaN coupling channel for Al compositions of 0.1 and 0.3.

Figure 8 illustrates the electric field (V/cm) along a cutline through the center of the gate region for two structures: (a) a conventional AlGaN/InGaN/GaN/AlGaN/SiC HEMT with an InGaN/GaN coupling channel, and (b) a hybrid AlGaN/CdZnO/GaN/AlGaN/SiC HEMT with a CdZnO/GaN coupling channel for Al compositions of 0.1 and 0.3. The electric field magnitude increases significantly in both structures as the Al content rises. For x = 0.1, the HEMT with a CdZnO channel exhibits a lower electric field than the HEMT with an InGaN channel. In contrast, at x = 0.3, the electric field in the HEMT with a CdZnO channel is considerably higher than in the HEMT with an InGaN channel. This suggests that at lower Al content, the HEMT with a CdZnO channel has a higher breakdown voltage, while at higher Al content, the breakdown voltage of the HEMT with a CdZnO channel decreases.

## 4. Conclusions

In conclusion, the incorporation of a CdZnO layer in AlGaN/GaN HEMT structures shows promise for enhancing device performance, particularly in terms of breakdown voltage and drain current. These improvements make CdZnO/GaN-based HEMTs highly suitable for high-power, high-frequency applications such as power amplifiers, RF transmitters, and radar systems. Future research should focus on optimizing the CdZnO layer’s thickness and exploring its thermal properties to fully unlock the potential of this hybrid structure. Additionally, further studies on the long-term reliability and scalability of CdZnO-based HEMTs will be essential for their adoption in commercial applications. GaN and its related compounds are commonly grown on substrates such as silicon carbide (SiC) or sapphire. However, recent efforts to fabricate HEMTs on silicon (Si) substrates are drawing interest due to the potential for cost reduction and compatibility with larger wafer sizes. We believe that the present device can be effectively adapted for use on Si substrates, aligning with these industry trends.

## Data Availability

The datasets used and/or analyzed during the current study are available from the corresponding author on reasonable request.
